# Supplementary material for: Patterns of Intron Gain and Loss in Fungi
Source: PLoS Biol. 2004 Nov 30;2(12):e422. doi: 10.1371/journal.pbio.0020422 (PMC532390; doi:10.1371/journal.pbio.0020422)
Supplement: Table S1 — Also available at http://genes.mit.edu/NielsenEtAl/. (4.3 MB ZIP). [file pbio.0020422.st001.zip › NielsenEtAl/html/515.html]

AN8280.1.NCU01654.1.MG07197.1.FG01419.1


```
 CLUSTAL W (1.82) Multiple Sequence Alignments - Introns Inserted


Sequence 1: NCU01654.1	692 aa
Sequence 2: MG07197.1	707 aa
Sequence 3: FG01419.1	708 aa
Sequence 4: AN8280.1	1003 aa
Alignment Length: 1005 aa
Number Identitical Residues: 241 aa
Alignment Score (without introns) 15109


MG07197.1 	MAPPKYIDDESFVKWTSEQPPPGAPYALPVPNTQRPNRTPVYRHWKLTDGPLISRFSDK~
NCU01654.1	MAPSQ--DTAAYIKSIAEPPPPGTPYALPVPGTQRPNRTAVYRHWKFVNSPLLETLDPA~
FG01419.1 	MALQTQKDYDIQNKKLFTVPPPGRPYGVPVAGTERPNRTAAYRHWAVGDGPLVTSLEPG~
AN8280.1  	MFFTQERAHYARAAELTQEPPKGKPYSVAIPGTEKDGRSPIYRSWWT-QKELVKTLDPK0
          	*                  ** * **.:.:..*:: .*:. ** *   :  *:  :.   

MG07197.1 	IRTMHEVFEESAKKHANKKFLGWRAWNPEKRAHEPKYTYMTYAEGAERRKNIGAGVVELH
NCU01654.1	HQTVHDLFEASVEKVPNKKCLGWRPWNAATKTYEPKYVWITYKEVAERRKNFGAGIVAIH
FG01419.1 	INSTHDILNRSARKWPNSKCLGTRHWNQATQQWEDKYDWITYADFDVRRKNFGAGLVEIH
AN8280.1  	VTTAHEIFESTANDTPKADCLGWRPYDPVKKTWG-QYQWISYGTVQKRRTDFGAGLVELH
          	  : *:::: :... .: . ** * ::  .:    :* :::*     **.::***:* :*

MG07197.1 	KRIGVT-ADKYGVGLWAPNCPQWQLFD~LGCISQSLWSVSLYETLGPETSEYIINHSELV
NCU01654.1	KSIGFT-EEKYGV-------------E1LALLSQSLFPISLYETLGPETTEYIINHSGLT
FG01419.1 	KGIGYA-SEKYGVGLWSQNRAEWQIAD1FGAASQSLYSVSLYETLGPDTTEYIINHAEVA
AN8280.1  	HKHNCARPGQYGIGLWCQNRPEWQITD1LACMSQSLYSVSIYDVLASDATQYIINHAELS
          	:  . :   :**:.  . . .. .  : :.  ****:.:*:*:.*..::::*****: : 

MG07197.1 	CVASSLPHIPQLIKLAARTPGLKMIISLDPLDNGEPASNTKLALLNQMAADAGIQIFSLE
NCU01654.1	AIACSVPHIPTLLKVAPRCPTLKLIISLDPLDAGEPAGHTKRELLNAAASNVGIKIFSID
FG01419.1 	CVVCSLPHIPVLLKMSPRLPGLKLIVSLDPLEQGELASHTKASVLNEIASHHGIQIFSMA
AN8280.1  	CVVASLPHIPTLIKLKPVLPNLKIIVSLDSLEAGEPAGHSKRALLESMAAGHDLTIYSMD
          	.:..*:**** *:*: .  * **:*:***.*: ** *.::*  :*:  *:  .: *:*: 

MG07197.1 	QVEAIGAQSGRPMRPPHEDDIITINYTSGTTGNPKGVVLKHSHGVAAIYAAISMGQFRNG
NCU01654.1	EVEALGVKSGLSMKPPKREDVLTINYTSGTTGDPKGVLITHAQGVAGITAARSMQSILSS
FG01419.1 	QVEEIGAKSGRAPQPPTREDICTINYTSGTTGNPKGVMITHGNAVSAIAGGRTNGNVCPK
AN8280.1  	QVEALGAASNRPLNPPKPSDIVTINYTSGTTGAPKGVVLTHENAVAATSGALVSVSQARG
          	:** :*. *. . .**  .*: ********** ****::.* :.*:.  ..    .    

MG07197.1 	DHHISYLPLAHIYGRMQDQLSFAIGGTTSFFRGDVLGLVEDLKLVRPNYFCSVPRLYNRF
NCU01654.1	DIHISYLPLAHIYGRMADQTALAEGASIGYFHGDIAGLVEDMKILRPTGLMSVPRLYNRI
FG01419.1 	DVHMSYLPLAHIYGRLIDQIAVAEGAAIGFFRGDILGLVDDMKILKPTGFISVPRLFNRF
AN8280.1  	DTFPSYLPLAHIYERLTEHAALWSGARIGYFHGNILELVDDLKVLKPTGFVSVPRLYSRF
          	* . ********* *: :: :.  *.  .:*:*::  **:*:*:::*. : *****:.*:

MG07197.1 	AQSIRIATTESQGFKGSMARHIVDAKKASMKLP-PGQATNKQWFYDRIWSPKVRQAVGLD
NCU01654.1	SGAVQAATIDADGFKGTLSRYVVEAKKQSMALP-TGKATNKHFLYDRIWTKKVLKGVGLD
FG01419.1 	NSAIRTATIEADGVRGALSRRVIDTKKANMRLP-PGQASNTHFLYDRIWTPKVKAAVGMD
AN8280.1  	GTAIRAATIEAPGFRGTLSKHIVAAKSANLKNPDPSKATVKHALYDRIWAKKVAAAVGLE
          	  ::: ** :: *.:*:::: :: :*. .:  *...:*: .: :*****: **  .**::

MG07197.1 	RARFMISGSAQLDPDVQEFLRAAFANDFFQGFGMTETYGNGTVQQPGDLTLGILGGPCVA
NCU01654.1	RARTMVSGSAQLDPEVHQFLRAAFGNDFIQGFGMTESYATGTAQVPGDFSTGNIGPPCPC
FG01419.1 	RVHSMVSGSAQLDPDVQQFLRAAFANHFAQGFGMTETYAVGSIQARGDFTTGNIGGPMCC
AN8280.1  	NAKQLISGSAPLDPSLHDFLRVAIGADFSQGYGLTETYAMACAQSPKDVTSGNCGRVAPC
          	..: ::**** ***.:::***.*:. .* **:*:**:*. .  *   *.: *  *    .

MG07197.1 	MEYCVESVPDLEYTIDDKQGPRGELLVRGPTVFSEYYKNPEETAKTVEADGWFHTGDIVQ
NCU01654.1	VELCIESVPDYDYTVDDKPNPRGELLMRGPIMFKEYYKNPEETAKVVEADGWFHTGDIVE
FG01419.1 	VELCLESVPEFDYTVDDKPNPRGELLIRGPVIFREYYKNDEETRKTLDADGWFHSGDICE
AN8280.1  	TEVCLLSLPDMEYSVDDKPFPRGELLVRGPNVFKEYLKNPEETSKAITEDGWFRTGDVAK
          	 * *: *:*: :*::***  ******:*** :* ** ** *** *.:  ****::**: :

MG07197.1 	VDKLGRIQIIDRKKNVLKLAQGEYISPERLENVYMGSSNLIATAFVHGDGKESTLVAVFG
NCU01654.1	VDSMGRFKIIDRKKNVLKLSQGEYISPERIENVYMASTNLIAMAFVHGDPKESSLVAIFG
FG01419.1 	VDKMGRFKIIDRKKNVLKLAQGEYISPERIENVYMGSTSLVNAAFVHGDGTQSSLVAIFG
AN8280.1  	IDDMGRIIIIDRRKNVLKLAQGEYISPERLEGIISSELGYLAQVYVHGDSMQTFLVAIFG
          	:*.:**: ****:******:*********:*.:  .. . :  .:****  :: ***:**

MG07197.1 	VDPEHFAPFASKIMGKTIAPTDAEGIKQACGDAKVMKQFMKHIDEIGRSHKFNSYEKVKA
NCU01654.1	IDPVTFAPYASRILKKNIAPENLEELKQSANDPRVKTAFLKLLDGIGKNHKFNSYEKVKN
FG01419.1 	IDVENFAPFASKILQETITPNQVADLRVAANNPKVKAKFLKVLDGIGKKHKFNSFEKVRN
AN8280.1  	VQAELFASFASKVLGRTIDPTDVAGIKAVLQDNKIKKAVLRDLERVAKKNKLAGYERVKN
          	::   **.:**::: ..* * :   ::    : ::   .:: :: :.:.:*: .:*:*: 

MG07197.1 	AHLALDPFSVENGIFTPT2LKLKRPQAARAFRSEIDRMYAEINSQP-VAKAKL~------
NCU01654.1	VYLDIEPFTIENELLTPT2LKLKRPQAARKFRAEIDRMYEEINAAA-QTKAKL~------
FG01419.1 	VHLDIDPFTIDNGIFTPT2LKLKRPQAAKAYREHIDRMYEELAAQEPIGKNKL~------
AN8280.1  	CALMVEPFSVENDLLTPT2LKLKRPPVVKKYRQLLDQLYEEANNEQSAPKAKL2EFPPDG
          	  * ::**:::* ::*** ****** ..: :*  :*::* *     .  * ** . ....

MG07197.1 	-----------~------------------------------------------------
NCU01654.1	-----------~------------------------------------------------
FG01419.1 	-----------~------------------------------------------------
AN8280.1  	GRHLHDLQNFP0NHVRVDPNNSHRSKMHLINALIFLVLLVHPALSSKFTITIPPSPVLPN
          	.    . .. . .    ....:  :.    .:         .: ::. : : ..:.  ..

MG07197.1 	------------------------------------------------------------
NCU01654.1	------------------------------------------------------------
FG01419.1 	------------------------------------------------------------
AN8280.1  	PQSLPADTHATLTSTTLARPLKAPLTRSATFVFSDFAAAECKDPSQHSISGSYLLDIRSA
          	..: .:.: :: :::: : . .:. : :::   :. :::.....:. : :.:   .  ::

MG07197.1 	------------------------------------------------------------
NCU01654.1	------------------------------------------------------------
FG01419.1 	------------------------------------------------------------
AN8280.1  	EYVFPPLRVDLDERGEVKGVWETFRGNEWGNRGAEKYIRPSGAQVGGGTGDVVVEARPVG
          	.   ..   . .. .. ..  .:  ... .. .:..   .:.:. ...:..   .: . .

MG07197.1 	-----------~-------------------------------~----------------
NCU01654.1	-----------~-------------------------------~----------------
FG01419.1 	-----------~-------------------------------~----------------
AN8280.1  	RKGFYEVRQTF1SPLSLLKNPMILLALVALAFTFGMPKLMENM1DPELREEFEKQSRASP
          	 ..  .  .:  :. :  ...    :  : : : . ..  ..  ...  .. ...: ::.

MG07197.1 	----------------------------------------------------
NCU01654.1	----------------------------------------------------
FG01419.1 	----------------------------------------------------
AN8280.1  	IAGAQSAMAGGGPANFDLAGWMAGAAPRGQPVPVSGSGAASGRENRGGARRR
          	 :.:.:: :....:. . :.  :.::. ... . :.:.:::. .. ..:
```
